# Contribution of the immune system to follicle differentiation, ovulation and early corpus luteum formation

**DOI:** 10.21451/1984-3143-AR2019-0087

**Published:** 2019-10-23

**Authors:** Noof Abdulrahman, Trudee Fair

**Affiliations:** School of Agriculture & Food Sciences, University College Dublin, Belfield, Dublin 4, Ireland.

**Keywords:** bovine, ovulation, corpus luteum

## Abstract

Much of what we know about the involvement of the immune system in periovulatory follicle differentiation, ovulation and subsequent formation of the corpus luteum in cattle is drawn from the findings of studies in several mammalian livestock species. By integrating published histological data from cattle, sheep and pigs and referring back to the more comprehensive knowledge bank that exists for mouse and humans we can sketch out the key cells of the immune system and the cytokines and growth factors that they produce that are involved in follicle differentiation and luteinization, ovulation and early follicle development. These contributions are reviewed and the key findings, discussed.

## Introduction

Optimum fertility underlies all livestock production systems and by it’s nature reflects the metabolic and immunological health status of the animal. Exposure to environmental insults such as heat stress, undernutrition and drought, metabolic and pathogenic disease have well-documented negative consequences for female fertility (Fair, 2010). In dairy cattle the period from 3 weeks pre-calving to 3 weeks post-calving, known as the transition period, has been the subject of much focus and there is substantial scientific evidence that it exerts a profound effect on the animal’s metabolic, immune and endocrine systems. Transition dairy cows become immunosuppressed due to lower dry matter intake, increased exposure to bacteria, and increased non-esterified fatty acid, beta-hydroxybutarate, concentrations and therefore more susceptible to increased incidence of endometritis and metritis, generally associated with reduced productivity and poor fertility in the rebreeding period (Sheldon *et al*., 2009; Thatcher *et al*., 2010; Giuliodori *et al*., 2013). Oocyte quality is considered a major contributor to the low fertility of these animals (Fair, 2010; Leroy *et al*., 2015), but so too is corpus luteum (CL) function (Niswender *et al*., 1994) and the endometrial environment. If we consider the ontogeny of the CL and it’s primary function when formed, it is obvious that these key contributory factors are intricately related. Moreover, numerous studies have outlined an integral role for immune cells in follicular development (Fukumatsu *et al*., 1992), steroidogenesis (Petrovská *et al*., 1996), ovulation (Brännström and Enskog, 2002) and CL formation and regression (Pate *et al*., 2010). Thus it is likely that as the immune and endocrine systems coordinate the normal development and functioning of these tissues (Hansen *et al*., 2010), their susceptibility to modulation by adverse metabolic and environmental environments will act as the primary conduit by which oocyte quality and CL function will be compromised. Taking this statement as our hypothesis, the aim of this manuscript is to review the molecular and cellular involvement of the cow’s immune system in follicle differentiation, ovulation and corpus luteum formation.

## Follicle differentiation and luteinization

Differentiation of the dominant follicle is associated with granulosa cell proliferation, increased intrafollicular concentration of estradiol (E2) and a switch from follicle stimulating hormone (FSH) to luteinizing hormone (LH)- responsiveness as they develop. Following the preovulatory gonadotropin surge, these estrogen-active follicles lose their capacity to produce E2, for detailed information see the excellent review by Ireland *et al*., (Ireland *et al*., 2000). The subsequent switch from E2 dominance to progesterone (P4) dominance in the follicular fluid of preovulatory follicles in the period between the LH surge and ovulation signals the onset of follicle luteinization (Dieleman *et al*., 1983). Pre-ovulatory follicle differentiation and luteinization appear to be characterized by an immune-cell specific temporal influx of leukocytes likely initiated in response to the high E2 concentration and various other chemoattractant cues produced by the developing follicle (Townson and Liptak, 2003). Histological analysis of dominant follicles from cattle, revealed that the first influx of cells is constituted by granular leukocytes, primarily mast cells, which infiltrate the theca layer of the follicle. Based on findings from sheep and pigs, it has been proposed that the mast cells in the theca layer become activated, likely in response to the LH surge and release the contents of their granules. Mast cell granules contain many factors, of which it is likely that tumour necrosis factor-alpha (TNF-α), recruits additional granular leukocytes such as eosinophils and neutrophils (Murdoch and Steadman, 1991; Standaert, *et al*., 1991). Following the peak in oestradiol concentration in the differentiated dominant follicle, the final phase of leukocyte infiltration, an influx of phagocytic monocytes occurs more or less in parallel with ovulation (Murdoch and Steadman, 1991; Standaert, *et al*., 1991).

At the molecular level, several reports have detailed the transcriptomic profile of ovarian follicle development in cattle: (Li *et al*., 2009; Gilbert *et al*., 2011; Walsh *et al*., 2012a; Christenson *et al*., 2013; Hatzirodos *et al*., 2014). Deep sequencing analysis of bovine follicular theca and granulosa tissue during pre-ovulatory follicle development, have revealed dynamic expression of many genes within immune-related pathways according to the stage of follicle development. Pathways associated with cell proliferation, tissue vascularization and angiogenesis were overpopulated during follicle differentiation (Walsh *et al*., 2012a), these processes are understood to be carried out by macrophages in the theca layer of the differentiating follicle (Fraser, 2006; Turner *et al*., 2011). Following the surge in the pituitary gonadotrophin LH, pre-ovulatory follicle development is directed away from differentiation and towards luteinization, initiating the earliest stages of CL development (Richards *et al*., 2008). In particular, the LH surge sharply increases the local production of the 2 angiogenic factors, basic fibroblast growth factor (FGF) 2 (Berisha *et al*., 2006) and vascular endothelial growth factor (VEGF) A (Schams *et al*., 2001), as the integrity of the basement membrane between the theca and granulosa-cell layers breaks down (Dieleman *et al*., 1983), the movement of leukocytes from the theca layer into the granulosa tissue is permitted and angiogenesis, required for CL formation is initiated.

## Ovulation

The biochemical events of mammalian ovulation have been likened to an acute inflammatory response, owing to the participation of leucocytes, classical inflammatory mediators as well as proteolytic enzymes (Richards *et al*., 2008). Based on data from studies in mice it is accepted that the initiation of the ovulatory process occurs primarily in granulosa cells (Richards *et al*., 2002; 2008). Indeed, the gene expression profile of bovine peri-ovulatory granulosa-cells is enriched with factors involved in acute inflammation and immunosurveillance (Walsh *et al*., 2012b). The expression of these factors by the granulosa cells may activate the ovarian innate immune system (Spanel-Borowski, 2011), as evidenced by the detection of acute phase proteins, defensins, interleukins and prostaglandins (PGs) in the pre-ovulatory follicular tissue and fluid (Takeda, 2004; Angelucci *et al*., 2006; Poulsen *et al*., 2019). Based on findings in rats, it is proposed that PGs and leukotrienes stimulate the synthesis and activity of collagenases which act to promote the degradation of the follicle matrix (Murdoch and Gottsch, 2003). While some of these factors may be expressed or secreted by the immune cells immigrating from the theca layer, it is likely that the damaged granulosa cells actively or passively release tissue damage signals initiating a pre-ovulatory inflammatory cascade. Typically an inflammatory cascade involves the activation of endothelial cells and local leucocytes, leading to the recruitment and accumulation of additional leucocytes, vascular endothelial cells and plasma proteins. Within the peri-ovulatory follicle, the cytokines TNF-α and interleukin-1 (IL-1) promote inflammatory associated processes, such as an increase in intrafollicular pressure, the activation of proteolytic pathways, collagenases and initiation of angiogenesis and tissue destruction and repair. These processes lead to the reorganization of the follicular stroma, extensive remodelling of the extracellular matrix and loss of the follicle’s surface epithelium, which result in a weakening in the follicle so that finally the follicle ruptures, expelling the cumulus enclosed mature oocyte and the follicle is transformed into a wound-like structure, the corpus hemorrhagica.

### Oocyte maturation

Ovulation is initiated by the LH surge which acts directly on the theca and mural granulosa cells of the follicle. However, the preovulatory oocyte does not express the LH receptor, and expression in the surrounding cumulus cells is very low (Lawrence *et al*., 1980), therefore the propagation of the LH stimulus throughout the follicle to the oocyte is mediated by secondary molecules, which initiate oocyte maturation. Oocyte maturation comprises expansion and proliferation of the cumulus cell layers, oocyte nuclear maturation, i.e. dissolution of the nuclear membrane and the resumption of meiosis, reorganization of the oocyte cytoplasmic organelles and a dramatic increase in protein synthesis and activation of molecular pathways (Fair, 2003; 2010). The propagators of the LH signal in the preovulatory follicle have been identified as three members of the epidermal growth factor (EGF)-family, the EGF-like peptides amphiregullin (AREG), epiregulin (EREG) and beta-cellulin (BTC) (Park *et al*., 2004). The ovulatory LH surge induces an acute upregulation of the EGF signalling network in mural granulosa cells, which is transmitted to the cumulus cells. This leads to initiation of mural granulosa cell luteinisation, production of an extensive extracellular matrix by cumulus cells and the closure of gap junctional communication between the oocyte and the cumulus cells. At the same time, the meiotic inhibitory signalling network mediated by C-type natriuretic peptide (CNP) and cyclic guanosine monophosphate (cGMP) in mural granulosa and cumulus cells is downregulated, leading to oocyte meiotic maturation (see Richani and Gilchrist for review) (Richani and Gilchrist, 2018). It is noteworthy that the amplification and propagation of the EGF signal from the mural to cumulus cells is dependent on the LH-induced production of PGE_2_ in mural granulosa cells (Shimada *et al*., 2006). Furthermore, oocytes are not directly responsive to EGF-like peptides (Conti *et al*., 2006; Park *et al*., 2004), therefore, the LH/EGF-peptide ovulatory signal is transmitted to the oocyte via the EGF receptor on cumulus cells. It has been hypothesized the acquisition of EGF signalling capabilities by the mural granulosa and cumulus cells is important and likely to represent a milestone in oocyte development and acquisition of competence (Ritter *et al*., 2015).

## Corpus Luteum Formation

The corpus hemorrhagica arises from the collapsed post-ovulatory follicle. Morphologically it resembles a fresh wound, but it is actually heterogeneous in nature, composed of multiple, distinctive cell types including steroidogenic cells, large and small luteal cells, which originate from the granulosa and thecal cells of the follicle ruptured at ovulation, as well as resident and migrating vascular endothelial cells, fibroblasts and immune cells (Lobel and Levy, 1968; Lei, *et al*., 1991; Spanel-Borowski *et al*., 1997; Penny *et al*., 1999; Bauer, *et al*., 2001; Davis and Pate, 2007).

### Immune system in the developing CL

Immediately after ovulation, in parallel with the onset of the differentiation of the follicular steroidogenic cells into luteal cells, resolution of the inflammation and consequential tissue damage must occur. This is initiated by immune cells recruited during ovulation (Murdoch and McCormick, 1993; Oakley *et al*., 2010; Watanabe *et al*., 1997; Gaytán *et al*., 1998). Chemoattractant cytokines (e.g., chemokines IL-8 and C-C motif ligand 5 and 2), produced by the endothelial, fibroblast and immune -cells, establish concentration gradients within the CL, which recruit and direct immune cell migration, primarily granulocytes, neutrophils and eosinophils that have originated in the spleen (Penny *et al*., 1999; Lobel and Levy, 1968; Spanel-Borowski *et al*., 1997; Jiemtaweeboon *et al*., 2011; Shirasuna *et al*., 2012). Immune cell migration is also enabled by the expression of ligands on immune cells, which interact with adhesion molecules on endothelial cells. Eosinophils are recognized as actors in the innate immune response to parasitic infections, asthma, and allergic conditions. Therefore, it is interesting to note that there is a rapid influx of eosinophils into the CL shortly after ovulation in cattle (Reibiger and Spanel-Borowski, 2000). The expression of P-selectin on endothelial cells appears to recruit eosinophils into the developing CL (Aust *et al*., 2000; Rohm *et al*., 2002). The arrival of these first responders, appears to be an important, but not essential, stimulus for angiogenesis during the early stages of CL development, as dexamethasone- induced eosinopenia resulted in lowered plasma P4 concentrations and reduced CL VEGFA protein production in cattle (Kliem *et al*., 2013). Moreover, the role of eosinophils appears to be restricted to the repair of the site of follicle rupture and early CL development as they are barely detectable later in the oestrous cycle when the CL is well established, or at the end of the cycle during CL regression (Reibiger and Spanel-Borowski, 2000; Rohm *et al*., 2002; Jiemtaweeboon *et al*., 2011).

Similar to eosinophils, neutrophils are important in the primary, nonspecific stages of acute inflammatory reactions and were also observed in large numbers, along with a high level of IL-8 (a potent neutrophilic chemoattractant), during the early luteal phase (d 1–4 of the estrous cycle) in the CL of cows (Jiemtaweeboon *et al*., 2011). They too appear to be integral to the resestablishment of the local microvasculature and the promotion of the acute inflammatory cascade, as both PMN and IL-8 were reported to induce angiogenesis in vivo (Koch *et al*., 1992; Komatsu *et al*., 2003) and in vitro (Schruefer *et al*., 2005). These findings were verified in bovine CL tissue in a series of in vitro experiments, where Jiemtaweeboon *et al*. (2011) demonstrated that supernatant from cultures of early CL tissue could induce PMN migration in vitro and increase PMN IL-8 production. Moreover, IL-8 stimulated endothelial cells of the CL to form capillary-like structures, indicating that IL-8 acts as a major PMN chemoattractant and a strong stimulator of angiogenesis in the early CL (Jiemtaweeboon *et al*., 2011). The concept of functional polarization of neutrophils (classic proinflammatory versus novel anti-inflammatory) has been proposed to explain their action in angiogenesis (Fridlender *et al*., 2009). Concomitant with vascular angiogenesis, macrophages and endothelial cells infiltrate the developing CL. The number of macrophages and monocytes in the CL increases during the early stages of development in cows, but they are substantially fewer in number compared to during CL regression (Penny *et al*., 1999; Lawler *et al*., 1999; Townson *et al*., 2002). In response to local cytokines and other signals, macrophages differentiate to acquire a functional phenotype that is specific to the requirements of the tissue. Within the developing CL, these cells produce and secrete various cytokines, such as TNF-α, interferon gamma, interleukins, PGs and angiogenic growth factors (Sakumoto *et al*., 2000; Townson and Liptak, 2003). The cytokine, TNF, is a potent stimulator of luteal PGs including PGF2α, PGE2 and PGI2 (Benyo and Pate, 1992; Sakumoto *et al*., 2000). Thus, TNF-α and TNF-induced PGE2 have been proposed as key regulators of CL vascularization (Okuda and Sakumoto, 2003; Korzekwa *et al*., 2008). A defined role for macrophages in promoting the vascularization of the developing CL is further substantiated by the findings of conditional macrophage ablation studies in mice, where it was shown that the ablation of macrophages in the early CL disrupted the ovarian vasculature and CL integrity (Turner *et al*., 2011).

There is very little evidence to suggest an essential involvement of T lymphocytes in the repair of the ovulatory site or the formation of the new CL. In fact, reports from several species, including bovine (Penny *et al*., 1999), buffalo (Ramadan *et al*., 2001), human (Best *et al*., 1996), pigs (Standaert *et al*., 1991) and sheep (Cavender and Murdoch, 1988), are equivacol in their descriptions of low numbers of CD4^+^and CD8^+^ T lymphocytes in early to late luteal phase CL tissue and infiltration of larger populations during CL regression.

While, the immediate response to follicle differentiation, E2 production, the LH surge and subsequent ovulation is characterized by large numbers of PMN and macrophages, a substantial influx of endothelial cells also occurs. Moreover, these endothelial cells form the greatest cohort ofproliferating cells in the early CL (Townson and Liptak, 2003; Reynolds and Redmer, 1999). Microvascular growth and development occur at an extremely rapid pace in female reproductive tissues and these tissues are highly vascular when mature. For example, most (~50–85%) of luteal cell proliferation occurs in the microvascular compartment (Reynolds *et al*., 1992; Reynolds *et al*, 1998). As a result, in the mature CL, microvascular pericytes and endothelial cells comprise 50–70% of the total cell population (Farin *et al*., 1986; Lei *et al*., 1991)

### Angiogenesis in the CL

In the ovary, the re-establishment of the luteal tissue microvasculature from pre-existing capillaries is a complex process that is necessary for the delivery of adequate levels of hormones and lipoprotein bound cholesterol into and out of the CL and ovary (Cherry *et al*., 2008) and is regulated by a number of growth factors. In cattle, peak expression of VEGF, its receptor VEGFR-2, FGF2, insulin-like growth factor (IGF), angiopoietin (ANPT) and hypoxia-inducible factor (HIF) family members has been reported from Day 0 - 4 of the oestrous cycle (Berisha *et al*., 2016; 2017; Castilho *et al*., 2019). The upregulation of these particular factors implies their particular importance for angiogenesis and maintenance of capillary structures during final follicle maturation and early CL development (Berisha *et al*., 2016). Luteal expression of VEGF occurs primarily in steroidogenic cells (granulose-lutein cells) and is regulated primarily by oxygen (Tropea *et al*., 2006). Hypoxia strongly induces angiogenesis, most likely through the HIF1–VEGF signalling pathway. Nitric oxide (NO) is produced by endothelial cells of luteal arterioles and capillaries; it is a potent vasodilator and stimulates endothelial cell proliferation, VEGF production and angiogenesis (Reynolds *et al*., 2000; Reynolds and Redmer, 1999). The purpose of luteal arteriole and capillary vasodilation is to facilitate increased blood flow and consequently delivery of peripheral immune cells to this site of tissue repair, regeneration and proliferation in the ovary. It is suggested that both FGF2 and VEGF play complementary roles in luteal angiogenesis (Robinson *et al*., 2009) as, FGF2 has also been shown to promote endothelial cell proliferation and appears to be critical to the initiation of the formation of the endothelial network in the bovine CL (Woad *et al*., 2009; Robinson *et al*., 2009). Furthermore, the suppression of VEGFA or FGF2 expression during the early luteal phase in cattle reportedly inhibited endothelial cell proliferation and reduced plasma P4 concentration (Kuhnert *et al*., 2008; Yamashita *et al*., 2008). A body of evidence also exists for a role for prostaglandins, including the luteolytic prostaglandin (PG) F2𝛼 in promoting CL vascularization and supporting CL growth. Indeed PGF2𝛼 has been shown to positively affect VEGF, FGF2, and P4 secretion in the bovine CL (Zalman *et al*., 2012; Miyamoto *et al*., 2010).

## Maintenance of the corpus luteum

The LH surge is the main trigger of ovulation and luteinization. Progesterone regulates the length of the estrus cycle by influencing the timing of the luteolytic PGF2α signal from the endometrium see review (Mishra and Palai, 2014). Furthermore, there is evidence to suggest that P4 may affect the secretory function of the bovine CL in a stage-dependent fashion, in an autocrine and paracrine manner that may be dependent on cell-to-cell contact and cellular makeup (Skarzynski and Okuda, 1999). For example, P4 affects the function of the early and mid CL in cattle (Skarzynski and Okuda, 1999; Duras *et al*., 2005), stimulating P4, oxytocin and prostaglandin secretion in the early CL, but later this is reversed as P4 inhibits PGF2a secretion in the mature CL. Recent studies have demonstrated that intra-luteal P4 is one of the most important factors supporting maintenance of the CL, acting to suppress apoptosis in bovine luteal cells through the inhibition of Fas and caspase-3 mRNA expression and inhibition of caspase-3 activation (Rueda *et al*., 2000; Okuda *et al*., 2004). Progesterone may also act to keep ovarian immune cells in check, by supressing T lymphocyte proliferation (Cannon *et al*., 2003).

## Corpus Luteum Regression

In the absence of an embryo(s) in the uterus, the process of CL regression begins on day 16 of the oestrous cycle in cattle (McCracken *et al*. 1999). Apoptosis of luteal cells and CL vascular regression are regulated by many different factors, however, in most species, uterine prostaglandin alpha (PGF2α) acts as the principal trigger for luteolysis. Although, it should be pointed out that there has been some debate about its direct action within the CL (Skarzynski and Okuda, 1999; Pate, 2003; Arosh *et al*., 2016). Nevertheless, PGF2α has been proven to acutely decrease P4 secretion by inhibiting 3ß-Hydroxysteroid dehydrogenase (3ßHSD) and steroid acute regulatory protein (StAR) mRNA expression and other rate-limiting steroidogenic enzymes in vivo (Tsai and Wiltbank, 1998; Atli *et al*., 2012), The process of luteolysis are understood to proceed with PGF2α induced angiolysis and vasoconstriction which limits the oxygen and nutrient supply to the tissue during luteolysis. Corpus luteum expression of members of the endothelin-1 (EDN1) system (EDN1, EDN converting enzymes, and EDNA and EDNB receptors) is up- regulated by PGF2 α (Mamluk *et al*., 1999; Klipper *et al*., 2010) during luteal regression (Klipper *et al*. 2004; Choudhary *et al*., 2005; Rosiansky-Sultan *et al*., 2006). Meanwhile mediators of PGF2 α luteolysis, i.e., vasoactive peptides, i.e. angiotensin II and atrial natriuretic peptide, trigger the luteolytic cascade, decrease blood flow and consequently inhibit P4 secretion (Shirasuna *et al*., 2004). Endothelin 1 is believed to participate in luteal regression, by promoting leucocyte migration and stimulating macrophages torelease cytokines, e.g. TNF-α and interferon-gamma (IFN-γ) (Girsh, *et al*., 1996); for review see also Smith and Meidan, 2014. Reportedly, TNF, TNF death receptors (TNF-RI), Fas and IFNγ mRNA expression is significantly increased during luteolysis in bovine CL (Korzekwa *et al*., 2008). Because of the ability of these cytokines to induce apoptosis in CL endothelial cells, they have been proposed as key regulators of bovine luteolysis (Okuda *et al*., 1999; Hojo *et al*., 2010). Additionally, cytokine membrane receptors, second messengers, including calcium ions and regulatory proteins are involved in apoptosis of steroidogenic and endothelial CL cells (Petroff, and Pate, 2001).

### Immune cells in CL regression

Luteal regression has been likened to an acute inflammatory process because of the short duration of luteolysis, the characteristic immune cell infiltration (neutrophils, macrophages, and T lymphocytes) and the dramatic change in vascular diameter and blood flow (Shirasuna *et al*., 2012). Many reports from several species describe an increase in lymphocytes or macrophages in the CL during luteolysis. Endothelial cell secretion of Monocyte chemoattractant protein 1 (also known as chemokine ligand 2 or CCL2), in direct response to TNF and IFN-γ stimulation, has been implicated in the recruitment of immune cells into the CL during luteal regression (Townson *et al*., 2002). In particular, macrophages and T lymphocytes, are proposed to play a central role in structural and functional CL regression (Best *et al*., 1996; Penny *et al*., 1999; Bauer *et al*., 2001; Townson, *et al*., 2002; Pate *et al*., 2010). The phenotypes of T lymphocytes resident in the bovine CL were previously quantified before and after the induction of luteal regression. Prior to regression, and in contrast to their ratio in peripheral blood, the proportion of CD8^+^-resident T cells was greater than CD4^+^-resident T cells, however there was no difference in the proportion of γδ+ lymphocytes in the CL compared to peripheral blood, nor was the proportion altered during luteal regression (Poole and Pate, 2012). The proportion of CD4^+^ Foxp3^+^ cells (i.e., T regulatory cells) was greater in a functional CL, compared to a CL induced to regress. This lead the Authors, to propose that Foxp3^+^ cells may control the actions of activated resident T lymphocytes to prevent premature luteal regression, but once luteal regression is initiated, a decline in the proportion the Foxp3^+^ cells weakens the inhibitory action on T lymphocytes, permitting their release of cytokines that may induce luteal cell death. In addition, the arrival of large numbers of monocytes, macrophages, and other cell types that create an inflammatory environment may augment the activity of the resident T lymphocytes.

It is truly remarkable that the activity of immune cells during luteolysis is confined to the CL and does not spread to the whole ovary, such tight control of inflammation ensures that CL tissue degradation remains localized with no effects on the surrounding tissue.

## Conclusion

It is well recognized that cattle require an appropriately functioning immune system for a swift and healthy recovery from parturition. The extent to which the maternal immune system is involved in bovine fertility is somewhat overlooked, yet it’s significant role in creating an appropriate microenvironment for final oocyte maturation, gamete transport and early embryo development reminds us that the immune system is intricately integrated in to the first stages of establishing pregnancy. Therefore, when seeking to optimize cow fertility, we should think first of the animal’s immune system and try to maintain its integrity, particularly in husbandry situations that expose the animal to significant metabolic and physiological stress.
